# Direct Determination of Moisture in Powder Milk Using Near Infrared Spectroscopy

**DOI:** 10.1155/JAMMC/2006/51342

**Published:** 2006

**Authors:** R. Nagarajan, Parul Singh, Ranjana Mehrotra

**Affiliations:** 1 Optical Radiation Standards National Physical Laboratory K. S. Krishnan Road New Delhi 110012 India

## Abstract

Moisture content in commercially available milk powder was investigated using near infrared (NIR) diffuse reflectance spectroscopy with an Indian low-cost dispersive NIR spectrophotometer. Different packets of milk powder of the same batch were procured from the market. Forty-five samples with moisture range 4–10% were prepared in the laboratory. Spectra of the samples were collected in the wavelength region 800–2500 nm. Moisture values of all the samples were simultaneously determined by Karl Fischer (KF) titration. These KF values were used as reference for developing calibration model using partial least squares regression (PLSR) method. The calibration and validation statistics are $R_{\;cal}^{2}:0.9942$, RMSEC:0.1040, and $R_{\;val}^{2}:0.9822$, RMSEV:0.1730. Five samples of unknown moisture contents were taken for NIR prediction using developed calibration model. The agreement between NIR predicted results and those of Karl Fischer values is appreciable. The result shows that the instrument can be successfully used for the determination of moisture content in milk powder.


Hindawi Publishing Corporation
Journal of Automated Methods and Management in Chemistry
Volume 2006, Article ID 51342, Pages 1–4
DOI 10.1155/JAMMC/2006/51342

### Direct Determination of Moisture in Powder Milk Using Near Infrared Spectroscopy

**R. Nagarajan, Parul Singh, and Ranjana Mehrotra**

*Optical Radiation Standards, National Physical Laboratory, K. S. Krishnan Road, New Delhi 110012, India*

Received 24 January 2006; Revised 6 March 2006; Accepted 18 May 2006

Moisture content in commercially available milk powder was investigated using near infrared (NIR) diffuse reflectance spectroscopy with an Indian low-cost dispersive NIR spectrophotometer. Different packets of milk powder of the same batch were procured from the market. Forty-five samples with moisture range 4–10% were prepared in the laboratory. Spectra of the samples were collected in the wavelength region 800–2500 nm. Moisture values of all the samples were simultaneously determined by Karl Fischer (KF) titration. These KF values were used as reference for developing calibration model using partial least squares regression (PLSR) method. The calibration and validation statistics are $R^2_{\text{cal}}$ : 0.9942, RMSEC : 0.1040, and $R^2_{\text{val}}$ : 0.9822, RMSEV : 0.1730. Five samples of unknown moisture contents were taken for NIR prediction using developed calibration model. The agreement between NIR predicted results and those of Karl Fischer values is appreciable. The result shows that the instrument can be successfully used for the determination of moisture content in milk powder.

Copyright © 2006 R. Nagarajan et al. This is an open access article distributed under the Creative Commons Attribution License, which permits unrestricted use, distribution, and reproduction in any medium, provided the original work is properly cited.

### 1. INTRODUCTION

Determination of moisture content in foodstuffs is very important as the moisture influences the shelf life of food products. Depending upon the water content, storage capacity of a food item varies. In addition, it influences the cost of transportation. Above all, moisture content determines how nutritive and tasty the food is. Hence, determination of moisture content becomes inevitable in food. Accuracy of moisture determination varies with the method employed. So, moisture analysis is still in a progress stage with various problems to be solved. There are various methods existing for determination of moisture content in foodstuffs that come under two major categories, namely, direct and indirect methods [1]. Out of these direct methods, Karl Fischer (KF) titration gets attention for moisture determination [2, 3]. Near infrared spectroscopy (NIR), out of all indirect methods, is extensively investigated for the determination of moisture content [4, 5].

Moisture analysis in milk powder has been an issue in terms of texture, flavour, shelf life, and so forth. Since it is an ingredient in many products, it should meet the required standard for customer satisfaction. Milk powder is hygroscopic in nature. Though moisture may not cause any health problems, grinding the powder (to avoid lump due to moisture) will result into change in flavour. Though different investigations are made on milk powder [6–12], attempts on analysis of moisture in milk powder are few [13–15].

The objective of the current research work is to assess the reliability of an indigenously built low-cost dispersive NIR spectrophotometer for quantification of moisture in commercial milk powder and to assess the possibility of employing the same process in dairy industry.

### 2. MATERIALS AND METHODS

Milk powder is one of the most suitable products to be analyzed using NIR spectroscopy, because of its uniform particle size and shape. Since milk powder has small particle size, it does not require any grinding. Milk powder of the same batch with different packing was procured from market for this study. One set of samples was prepared by drying milk powder for different timings to lower the moisture content more than the normally available level. Another set was prepared by conditioning the samples to constant relative humidity for different timings to increase the moisture level. Hence, a set of forty-five samples with a range of 4–10% moisture was obtained for analysis. Samples were well homogenized before experiments. Experiments were carried out within a very short span after sample preparation in order to minimize experimental errors.

ELICO SL-153 dispersive NIR spectrophotometer was used for recording the spectra. The instrument has grating with 300 lines/mm as monochromator. Bandwidth of the instrument is 20 nm. Ceramic block was used as reference for diffuse reflectance measurements. Spectra were recorded in the wavelength region 800–2500 nm under controlled room ambient. Sample accessory was washed with distilled water and dried completely to remove the existing moisture before loading the next sample. A reference spectrum was collected every time before scanning a new sample. Reference analysis was carried out on the same set of samples using automated Karl Fischer titrator (KAFI, Lab-India) for their moisture content. Different dissolution times were tried during KF measurements in order to optimise the results. Finally, stirring time of 6 minutes was selected to dissolve entrapped moisture present in the sample. For the KF titration, approximately 30 ml of absolute dry methanol (99.99% pure) was taken in titration vessel, and an accurately weighed sample was added to it. Then the titration was carried out with KF reagent (pyridine free) using automated KF titrator with 6-minute stirring time. The end point of the titration was determined by polarized double-pin electrode and the moisture value (in percentage) was displayed on the KF titrator screen. Solvent was replaced before every experiment. Analysis was randomly repeated for the same sample for cross checking the results obtained. The moisture values thus obtained by KF titration for forty samples were used to develop calibration model for the spectral measurements carried out. Five samples were considered as prediction set samples and their moisture values were predicted using developed calibration model.

GRAMS 32 software was used for data processing and developing calibration model for prediction process. All spectra were mean-centered before analysis. Necessary smoothing was done by box-car smoothing method. Savitzky-Golay polynomial filter was used with 13 data points to obtain second derivative spectra.

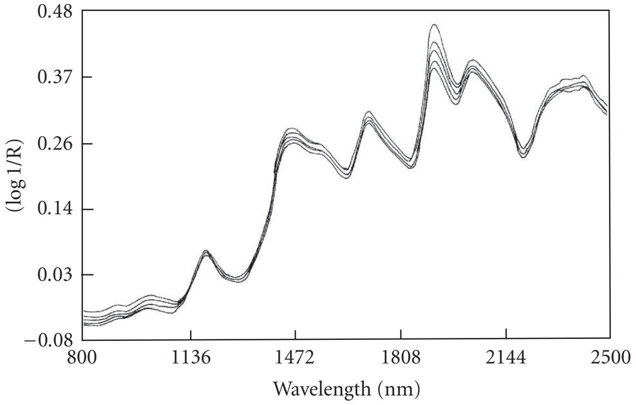

Figure 1: Overlaid spectra of milk powder samples with different moisture content in 800–2500 nm region.

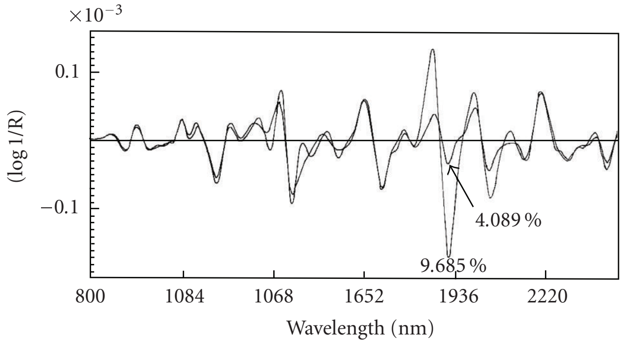

Figure 2: Second derivative spectrum of milk powder in 800–2500 nm region.

### 3. RESULTS AND DISCUSSION

The overlaid spectra of milk powder with different moisture content in the wavelength region 800–2500 nm are shown in Figure 1. The combination band of O−H stretch and O−H bending vibration mode is discernible as a strong absorption centerd at ∼ 1940 nm, in agreement with the value reported in literature. The O−H stretch first overtone appears as a medium absorption band at ∼ 1455 nm. The O−H stretch second overtone appears only as a weak band at ∼ 930 nm. In addition, the spectrum shows a medium strong band at ∼ 1720 nm and a weak band at ∼ 1200 nm, which is attributed to first and second overtone of C−H stretch, respectively. Absorption band at ∼ 2060 nm is attributed to the N−H stretch second overtone which is due to the presence of protein constituents in the sample. It is evident from Figure 1 that there is an increase in log (1/R) values with increasing moisture content in samples. Also, occurrence of a marginal shift at ∼ 1940 nm band towards higher wavelength with increasing moisture level was observed over the experimental results. This 1940 nm band may serve as a marker band for moisture analysis in milk powder. Absence of peak at this wavelength has also been considered as a proof for absence of moisture [10].

Since absorption bands in the regions 1425–1475 nm and 1900–1950 nm are stronger, they are often applied for quantitative analysis of moisture content. Figure 2 shows second derivative spectra of two samples with different moisture levels. It is maybe observed from Figure 2 that the variation in moisture content between the two spectra (4.089% and 9.685%) is easily observable at ∼ 1455 nm and ∼ 1940 nm. The peaks at ∼ 930 nm of OH absorption band show least variation in moisture level, which may not be so effective for calibration. Hence the spectral regions 1425–1475 nm and 1900–1950 nm were chosen for calibration.

Calibration was carried out by partial least squares regression (PLSR) method. The calibration statistics for two different wavelength regions are given in Table 1. It is clear from Table 1 that the model developed in 1900–1950 nm wavelength region has better correlation coefficient with low root mean square error of calibration. Validation was performed by cross-validation process, that is, leaving one sample of the calibration set at a time for prediction. The

Table 1: Calibration statistics for moisture in milk powder for different wavelength regions.

| Analyte | Wavelength region (nm) | PLS factor | Calibration | | Validation | |
|---|---|---|---|---|---|---|
| | | | $R^2$ | RMSEC | $R^2$ | RMSEV |
| Moisture | 1425–1475 | 5 | 0.9921 | 0.1250 | 0.9302 | 0.3430 |
| Moisture | 1900–1950 | 3 | 0.9942 | 0.1040 | 0.9822 | 0.1730 |

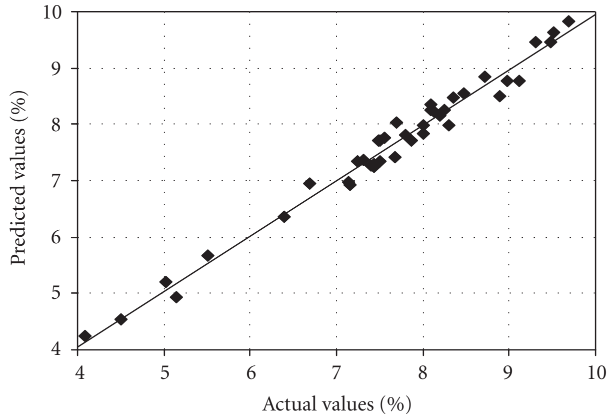

Figure 3: Actual (KF titration) versus predicted (NIR spectroscopic) values of moisture in calibration set.

actual versus predicted moisture values of calibration set samples are shown in Figure 3. The actual values (KF values) are plotted along $x$-axis, and NIR predicted values are plotted along $y$-axis. The line fit shows the closeness of data points, that is, the agreement between the actual (KF) and predicted (NIR) values of moisture in the calibration set. This may also be ensured by the calibration statistics given in Table 1. Figure 4 shows the score response of moisture for the first two principal components (PCs). Principal components are nothing but the projection of data through new variables. It is clear from the plot that each sample is distinct, but most of them set together due to closer moisture content values. The eigenvalues of the first, second, and third PCs are 96%, 2%, and 1%, respectively. Therefore these three components were used to develop calibration model. The PLSR loading vectors were strongly weighted towards the water absorbing NIR wavelengths, indicating that the PLSR calibration model mainly uses the information at these wavelengths. The first loading vector (in both cases: 1425–1475 nm and 1900–1950 nm) is almost identical to the spectrum of milk powder, and the following two loading vectors have prominent peaks in the water absorbing NIR region.

The concentration residual moisture values are shown in Figure 5. Concentration residual gives a clear picture about calibration. It explains the range of variation that the predicted concentration values exhibit for those samples present in calibration set. Moreover, the residual plot, with sample number versus, concentration residual values helps us to find out the deviation in concentration of each sample from its actual value. So, it acts as a tool to identify the outliers. It may

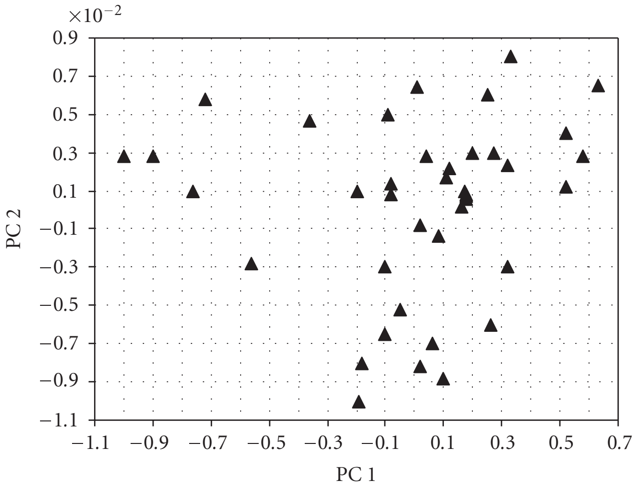

Figure 4: Score plot of PC 1 versus PC 2 for moisture in milk powder samples.

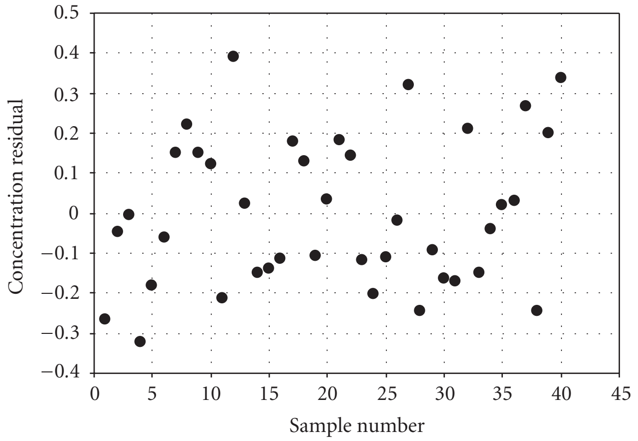

Figure 5: Concentration residual plot for moisture in calibration set.

be explicit from Figure 5 that most of the moisture residual values (around 85%) lie in the range of ±0.5.

A set of five samples was employed for prediction. Spectral measurements were made for these samples and their moisture values were determined using the developed calibration model. KF titration was performed for the same sample, set to estimate the moisture present. The NIR predicted values were compared to those of KF titration results. The comparison is given in Table 2. The agreement in results for both methods may be observed from the Table 2.

### 4. CONCLUSION

The reliability of Indian low-cost dispersive NIR spectrophotometer for the determination of moisture content in commercial milk powder has been explored. Forty samples with different moisture content were analyzed using NIR diffuse reflectance mode. Calibration and validation processes were

Table 2: Actual (KF titration) and predicted (NIR spectroscopic) values of moisture in prediction set.

| KF titration values (%) | NIR spectroscopic values (%) | Bias |
|---|---|---|
| 6.962 | 6.977 | 0.015 |
| 9.707 | 9.827 | 0.120 |
| 7.729 | 7.753 | 0.024 |
| 4.922 | 4.923 | 0.001 |
| 7.444 | 7.854 | 0.410 |
| $R^2 = 0.9345$ | | |
| RMSEP = 0.1915 | | |

performed by partial least squares regression method. Cross validation method was employed for validation. The calibration was carried out in the regions 1425–1475 nm and 1900–1950 nm. The calibration model developed in the region 1900–1950 nm was found to be better with correlation coefficient $R^2$ 0.9942 and RMSEC 0.1040. This calibration statistics shows the accuracy of the developed calibration model. Five samples of unknown moisture value (prediction set) were experimented to predict their moisture content. KF titration was also carried out for the same reason to examine the reliability and effectiveness of NIR analysis. Comparison of NIR spectroscopic results with those of KF titration yields interesting observations. The NIR spectroscopic and KF titration results were very close thereby indicating the accuracy of an Indigenously built low-cost dispersive NIR spectrophotometer for the determination of moisture content in milk powder, which may be employed in dairy industry for moisture analysis.

### ACKNOWLEDGMENT

R. Nagarajan is thankful to the Council of Scientific and Industrial Research (CSIR), India, for its support in pursuing the research work.

### REFERENCES

[1] H.-D. Isengard, "Water content, one of the most important properties of food," *Food Control*, vol. 12, no. 7, pp. 395–400, 2001.

[2] C. A. De Caro, A. Aichert, and C. M. Walter, "Efficient, precise and fast water determination by the Karl Fischer titration," *Food Control*, vol. 12, no. 7, pp. 431–436, 2001.

[3] H.-D. Isengard and P. Heinze, "Determination of the water content in different sugar syrups by halogen drying," *Food Control*, vol. 12, no. 7, pp. 483–486, 2001.

[4] P. C. Williams, K. H. Norris, C. W. Gehrke, and K. Bernstein, "Comparison of near infrared method for measuring protein and moisture in wheat," *Cereal Foods World*, vol. 28, pp. 149–152, 1983.

[5] M. J. Adams, K. Latham, N. W. Barnett, and A. J. Poynton, "Calibration models for determining moisture and fat content of processed cheese using near-infrared spectrometry," *Journal of the Science of Food and Agriculture*, vol. 79, no. 10, pp. 1232–1236, 1999.

[6] N. Özkan, B. Withy, and X. D. Chen, "Effects of time, temperature, and pressure on the cake formation of milk powders," *Journal of Food Engineering*, vol. 58, no. 4, pp. 355–361, 2003.

[7] B. R. Nielsen, H. Stapelfeldt, and L. H. Skibsted, "Early prediction of the shelf-life of medium-heat whole milk powders using stepwise multiple regression and principal component analysis," *International Dairy Journal*, vol. 7, no. 5, pp. 341–348, 1997.

[8] E. L. Celestino, M. lyer, and H. Roginski, "The effects of refrigerated storage of raw milk on the quality of whole milk powder stored for different periods," *International Dairy Journal*, vol. 7, no. 2-3, pp. 119–127, 1997.

[9] J. Štencl, "Water activity of skimmed milk powder in the temperature range of 20-45°*C*," *Acta Veterinaria Brno*, vol. 68, no. 3, pp. 209–215, 1999.

[10] T. K. Kockel, S. Allen, C. Hennigs, and T. A. G. Langrish, "An experimental study of the equilibrium for skim milk powder at elevated temperatures," *Journal of Food Engineering*, vol. 51, no. 4, pp. 291–297, 2002.

[11] H. Stapelfeldt, B. R. Meisen, and L. H. Skibsted, "Effect of heat treatment, water activity and storage temperature on the oxidative stability of whole milk powder," *International Dairy Journal*, vol. 7, no. 5, pp. 331–339, 1997.

[12] I. Ben-Gera and K. H. Norris, "Influence of fat concentration on the absorbtion spectrum of milk in the near infrared region," *The Israeli Journal of Agricultural Research*, vol. 18, pp. 117–124, 1968.

[13] S. Rückold, K. H. Grobecker, and H.-D. Isengard, "Determination of the contents of water and moisture in milk powder," *Fresenius' Journal of Analytical Chemistry*, vol. 368, no. 5, pp. 522–527, 2000.

[14] C. Reh, S. N. Bhat, and S. Berrut, "Determination of water content in powdered milk," *Food Chemistry*, vol. 86, no. 3, pp. 457–464, 2004.

[15] R. J. De Knegt and H. Van Den Brink, "Improvement of the drying oven method for the determination of the moisture content of milk powder," *International Dairy Journal*, vol. 8, no. 8, pp. 733–738, 1998.

